# A shared fate: adapting and personalising medical care from the perspective of a refugee reception country

**DOI:** 10.1186/s12992-022-00880-y

**Published:** 2022-10-21

**Authors:** Marcin Śniadecki, Zuzanna Boyke

**Affiliations:** 1 Chair of Gynecology, Obstetrics and Neonatology, Department of Obstetrics and Gynecology, Gynecological Oncology and Gynecological Endocrinology, Medical University of Gdańsk, M. Smoluchowskiego 17 Str, 80-214 Gdańsk, Poland; 2 Department of History, University of Gdańsk, Gdańsk, Poland

**Keywords:** War experience, Female refugees, Childhood disease, Migration, Healthcare system, Solidarity of women, Pregnancy, Art therapy, Burnout syndrome

## Abstract

The article outlines socio-medical challenges faced by a country hosting refugees. Since the full-scale Russian invasion, more than 3.6 million Ukrainians have fled to Poland. Among these, a 9-year-old post-transplant patient, Tetiana. The story of the girl and her family reveals the refugees’, the authors’, and other doctors’ varied attitudes towards the war. Considering differences between the Polish and Ukrainian health care systems, and the need for well-coordinated and effective assistance, a professional, and realistically financed system needs to be established. The presence of Ukrainian doctors is potentially advantageous because of the shared language with Ukrainian refugees. Notwithstanding the medical community’s demonstrable solidarity, the months of effort and the awareness that many people may not be helped may result in burnout. Supporting others starts with us, so that neither poor management, nor cultural differences or simple fatigue, undermine the community cohesion that Poles and Ukrainians can build together.

**Supplementary Information:**

The online version contains supplementary material available at 10.1186/s12992-022-00880-y.

One of the most important dates in the Polish calendar of national anniversaries is August 1. This is when the victims of the Warsaw Uprising of 1944 are commemorated. The memorial symbol is the sounding of alarm sirens throughout the country, punctually at 17:00 h. This year, on August 1, the sirens howled as usual, but not with a single voice. In many cities, the mayors protested that it was more important to care for those who have been with us for several months, escaping the hell of war, than it was to maintain the habitual national symbolism. “For Ukrainians, this sound is a reminder of trauma,” they said [1].

Although the discussion about sirens started in April, with the celebration of the anniversary of the Smolensk catastrophe [2], it intensified in August; and it is to be expected that this topic will recur in the lead-up to subsequent memorial dates. The problem is that this is not only a political or purely ad hoc discussion. A serious national debate is needed on the mental health, general well-being and, above all, the overall health of refugees from Ukraine. Such a discussion should be followed by systemic solutions that will make it possible to truly help the millions of people torn from their homeland. Russia’s aggression towards Ukraine and the resulting exodus of people from the East of Poland have also spotlighted the fact that both Polish and global health care systems were not adequately prepared for the challenges of this extreme and sudden displacement of the people.

When in early 2022, the severe acute respiratory syndrome coronavirus 2 (SARS-CoV-2) pandemic appeared to be the biggest global problem, a new threat emerged. In only the first seven days after the 24 February Russian invasion of Ukraine, over one million people fled from Ukraine, and of that number, 600,000 arrived in Poland (according to United Nations High Commissioner for Refugees data). This is the fastest mass exodus of people from a European country torn by an armed conflict since World War II. By the 20th day of the invasion, 3 million people had fled from Ukraine, bringing 1.8 million to Poland (United Nations data) [3]. At that time, the European Commission was estimating up to 7 million refugees could cross over, but that there could be more if the scale of hostilities continued or intensified [4]. Now, the number of Ukrainian refugees who have crossed the border into Poland has reached 5.4 million (as of August 16, 2022) [5].

These staggering numbers translate into concrete human dramas. 17 people, including pregnant women, were injured when a maternity and children’s hospital in Mariupol was bombed by the Russians. News media around the world circulated a photo of a young pregnant woman being stretchered out of the rubble by rescuers – the woman and her baby died of injuries – and that photograph has become a shocking symbol of the barbarity of this war (Additional file 1). Ukrainians often do not even have a chance to say goodbye to their dead – during the attack on Azovstal in Mariupol, ceasefires broken by heavy Russian shelling made it impossible to remove the bodies of the dead. Simply put, on the eve of the next anniversary of the Soviet attack on Poland which took place during World War II on 17 September 1939, the ruins of Mariupol and other Ukrainian cities razed to the ground and the spectacle of death and destruction cynically directed by Putin, form a tragically too real backdrop to any national wartime memorials.

In Albert Camus’ allegorical novel *The Plague* (1947), whose *Mise-en-scène* is a 19th century epidemic, one character says: ‘It’s impossible it should be the plague, everyone knows it has vanished from the West’ [6]. Echoing the sentiments of this expression in relation to the war in Ukraine, we want to show the extraordinary story of characters who are all too real in the current medical and social conditions. They are a contrast to the postulates of the medical community, formulated for years and described in prestigious magazines. These people seem to be saying “wait – I’m checking what have we done together as medics and as societies in terms of migration and health protection?”

## Connected by an allograft

Two girls, 9-year-old Tetiana from Ukraine and 5-year-old Marcelina from Poland, both suffer from atresia of the biliary tract with polysplenia and visceral inversion. Fate brought them together six years ago at the Children’s Memorial Health Institute in Warsaw, where they underwent a liver transplant. Now they have met again since Russia invaded Ukraine.

Tetiana’s family consists of her 24-year-old sister Yiuliia, in the 32nd week of her pregnancy, and her 48-year-old mother, Svietlana. When the war broke out, the girl’s father, Vlad, and Yiuliia’s husband Anatol remained in Ukraine to fight for their homeland. Tetiana’s transplant coordinator in Warsaw was concerned about the possible fate of the girl and her family and she persuaded them to flee to Poland. At the same time, the President of the Polish “For Life” Foundation, working on behalf of post-transplantation children, searched for a new home for them. It was then that families were brought back together by the allograft: Marcelina’s parents, when the war commenced, decided to host Tetiana’s refugee family in their apartment in Gdańsk. Unfortunately, when the family arrived, it was discovered that they suffered from several health problems that had not been well attended to in Ukraine (orthopedic and dental issues in Svietlana; gastritis, dental problems, and impaired vision in Tetiana; and growth retardation of the fetus was also suspected). The Polish woman Paulina, mother of Marcelina, who is equally burdened by the chronic illness of her own child, willingly agreed to host the refugee family because she understood their situation, one that is significantly worse than her own. In this position, Paulina has become a health visitor, social worker, and psychologist trying to secure appropriate care for her Ukrainian guests even while continuing with her everyday life and work commitments (Additional file 2). Paulina (Author [MŚ] had cared for her during her pregnancy) brought the Ukrainian family to me when they arrived in Poland and asked to examine the pregnant Yiuliia. Her fetus was developing normally. A week later, Yiuliia was seen by a public sector doctor, who, without examining her, referred her to a hospital that she did not have to go to.

There is no proper post-transplant care in Ukraine (including comprehensive blood testing, immunosuppression level management, fever management, and dental care). In Poland, in addition to helping with her underlying disease, Tetiana received dental care, a food allergy which was associated with abdominal pain was diagnosed, and she qualified for tonsillectomy. In addition, art therapy classes were organized for her. We decided that this method would be effective in the case of a child with post-war trauma. What’s more, therapy through art allowed us to break down some of the language barriers (Additional file 3) (ZB).

After a few days, Yiuliia’s husband decided to take his family back to the war-torn country. In this context, it is worth noting that officially, Ukraine has been asking refugees not to return yet, fearing that having fled once they will have to do so again. On the other hand, many refugees are also suffering from, and compelled by their Survivor Syndrome guilt [7].

## Help millions one person at a time

One of the key elements of support in a war situation is the provision of medical assistance. Shipments of medical supplies, ambulances, and specialist equipment from Poland to Ukraine have been ongoing. This humanitarian assistance helps non-government organizations (NGOs), foreign foundations (e.g., in oncology, St. Jude’s), the medical council, scientific societies, and hospitals.

The most vulnerable, namely pregnant women and children under 5, as well as the chronically ill (e.g., diabetics), are provided for first. The evacuation of vulnerable and seriously ill patients is complicated due to a variety of issues, including their clinical condition, the need to comply with regulations governing children crossing a border, difficulties for fathers related to the prohibition of men leaving Ukraine, and the ban on Ukrainian truck drivers from crossing the border into Poland.

During the first three weeks of the 2022 war, approximately 1,800 Ukrainians were admitted to Polish hospitals, and half of these were children, including patients requiring dialysis and cancer therapy. Alongside this, according to official figures, during the first months of the war Poles spent approximately PLN 10 billion (EUR 2.09 billion) from private funds to help Ukrainians, and almost PLN 16 billion (EUR 3.35 billion) from public funds [8]. Bank Pekao analysts estimated that the cost of hosting 2 million refugees from Ukraine may require government expenditure of PLN 24 billion (EUR 5.04 billion) during 2022 and 2023 [rate as on 23 August 2022].

Medical assistance has also been available online. Two doctors from Białystok set up a group called “Medics for Ukraine”, which connects practitioners from various medical professions [9]. The purpose of this initiative was to facilitate linking those people who need medical help with those who are willing and able to provide it (free of cost to patients). So far, over 16,000 medics have registered. Medical services have also been provided within Poland at border crossing points, along the ‘green corridors’ used by refugees on the Ukrainian side of the border, and doctors are offering free in-clinic consultations for refugees.

Notwithstanding the dramatic humanitarian needs arising from the war, the coronavirus disease 2019 (COVID-19) pandemic has not gone away and cannot be forgotten. Since the end of February 2022, Poland’s south-eastern border has seen a massive influx of people from a country where the COVID-19 vaccination rate (34.5%) has been one of the lowest in Europe.

## Fragile affections and a shared fate

No country can withstand the impact of such a rapid and massive migration without stress. Of first importance, is the need to work towards creating a system that could be resistant to all kinds of crises, that is operationally flexible, that can adapt to changing needs, and that is realistically financed and well managed. After all, we need to care for all those who have escaped the war, into Poland, and who are likely to remain in our country for some time. Both individual and groups of medical practitioners, as well as NGOs, must be able to meet the refugees’ health care needs in a more organised way than we did with Paulina and her family. Part of this is to develop a nationwide register of refugees who receive assistance, to know what kinds of skills and resources they need.

This problem does not only concern Poland. For years, researchers have emphasized the need for a coordinated global research agenda on migration and health. An efficient system would be to collect meaningful data on an ongoing basis, whose identification, customisation, and searchability would become crucial in enabling national and transnational health care systems to adapt and respond [10]. Much has been said about this as early as February 2017, during the 2nd Global Consultation on Migration and Health in Colombo, Sri Lanka, and in the months following those discussions. A global center of knowledge on migration and health is urgently needed, and the crisis we are currently witnessing in Poland is a prime example of this need. The beneficial uses of such a database would include the archiving of the rich experiences of various medical communities – in our instance the experiences gained by Polish medical professionals.

Secondly, there is no doubt that the visit of Ukrainian doctors to Poland may be extremely valuable in increasing the provision of medical care. Apart from the obvious reasons, such as the experience of Ukrainian medics, the primary reasons are practical – namely, the dramatic shortage of doctors and nurses in Poland. The government simplified the regulations to allow overseas medics to work in their fields in Poland, including from Ukraine; and more than 800 Ukrainian doctors have already recently applied to work in Poland [11]. So, what is the problem? Local governments complain that the approval process is overly bureaucratic, especially regarding the issuing of work permits by local medical chambers [12]. A solution is to automatically recognise the certified qualifications of medics from Ukraine, which is postulated in many circles [13]. To include these doctors and other medics in the Polish system, it is important to note that medical education in Ukraine differs significantly from the Poland system in many respects, including that Ukrainian specialization is for one to two years (compared with four to six in Poland), and that relatively few Ukrainian medics speak Polish, and vice versa. Nevertheless, granting official permission to work should not take the number of weeks it currently does, because this creates an unnecessary blockage in the path towards improving a system in crisis.

A related problem is the language barrier facing patients. Clerical obstacles often arise from a misunderstanding or lack of appropriate translation. Some refugees only have documentation in Ukrainian. Ukrainian medics can help solve the problem of language, but they also need the support of translators. Overall, language barriers are one of the main factors limiting refugees’ access to healthcare [14].

The third matter, and this is no less important than other needs during wartime, is that we must take care of ourselves and of our own society, because only then will we be able to help our guests from Ukraine. This sentiment can be seen in the article “Historical perspectives on xenotransplantation” by Schlich and Lutters in which the authors discuss how the history of medicine, and its socio-political contexts, touch human lives [15]. When we consider that the procedure of xenotransplantation has encountered a series of social obstacles that the authors call a form of “cultural rejection”, we can see there are likely to be parallels in the present social domain, between host and refugee. We are convinced that long-term assistance to refugees in Poland must begin with the Poles themselves. We cannot allow prejudices, a badly organized health care system, or an unfair distribution of financial resources to ruin the very community that we want to share and co-develop with our Ukrainian guests.

In Poland, there is a very long tradition of rapid “mass movements”, but that history is also full of the failures of such movements, many of them uprisings, not only because of external forces. Given this history, and the present context, medical practitioners must not forget about themselves. Long periods of providing voluntary help are exhausting both physically and mentally. The enormity of suffering and the prospect of failing to help everyone who needs help can lead to a feeling of hopelessness and burnout. That is why it is important to foster solidarity within the medical community, and tenderness, expressed as concern for others and the consciousness of a shared fate. As the Polish Nobel laureate (2018) Olga Tokarczuk once said: “Tenderness is the most modest form of love. […] Tenderness is spontaneous and disinterested; it goes far beyond empathetic fellow feeling. Instead, it is the conscious, though perhaps slightly melancholy, common sharing of fate” [16].

## Questions about agency and solidarity

Half a year after the outbreak of the war, it is also a good time to elevate the substantive discussion about Poland’s solidarity with its neighbour. It should be emphasized that thousands of refugees have also been received by countries such as Hungary, Romania, and Slovakia [5]. However, it is Poland that has taken the bulk of the responsibility for supporting people fleeing the war. In the long term and given the problems associated with an underdeveloped, professional support system for migrants, providing the care required may become an increasing challenge. One of the lessons of the coronavirus pandemic has been the importance of European and global solidarity. The migration crisis is a test of whether this solidarity can be reanimated transnationally to work for the common good [17].

An examination of the case of Tetiana and her family brings a bittersweet conclusion. Thanks to the involvement of many people, it was possible to help the sick child. We are aware that this is just one example; medics in Poland help hundreds of refugees every day, often devoting their private time and resources to it. This positive aspect, however, also raises serious questions and arrives at sad conclusions. Why were we not better prepared for such a crisis? Had we taken all possible measures to minimize the effects of the mass exodus? The UCL-Lancet Commission on Migration and Health, in its key message in 2017, called for migrants’ health to be addressed through improved leadership and accountability. Calls have been made for the UN to appoint a special envoy on migration and health, and for governments to establish national contact points. As we can clearly see today, this would enable much-needed coordination and ensure that migrants are included in all decisions about their health. Establishing a Global Observatory on Migration and Health that would develop an evidence-based indicator for better reporting and ease of monitoring, was also proposed [18]. Our story of one 9-year-old girl from Ukraine shows both the levels of goodness, efficiency, and solidarity that exist, as well as the neglect of a broader perspective. Though we are currently amid a migration crisis, we believe that this is the right time to ask questions about agency, consistency, and solidarity. We call on the entire medical community to join our appeal; and to heed our plea that the conversation about migrants’ needs is not limited to questions of symbolism but reaches into the substantive core of the problem.

## Postscript

The refugee family that we helped have returned to Ukraine. Yiuliia gave birth to a healthy boy on May 31, 2022. Our feelings about their decision match those expressed by Paulina in this message:

*I have mixed feelings. On the one hand, I understand it, because Mrs. Svietlana said that she had to plant vegetables so that they could eat all year round. On the other hand, I think it’s a stupid decision. After all, Yuliia could stay at least until the birth to be sure that the baby was healthy. I would not have gone back, if only because of the health care needed for Marcelinka.*

But the most important thing on our minds is that this decision did not have to be made by no one of us and we must accept it. This is their life and choice after all.

## Electronic supplementary material

Supplementary Material 1

Supplementary Material 2

Below is the link to the electronic supplementary material.

Supplementary Material 3

## Data Availability

Not applicable.

## Abbreviations

COVID-19: coronavirus disease 2019
NGO: non-government organization
SARS-CoV-2: severe acute respiratory syndrome coronavirus 2
