# Supplementary figures and images for: A shared fate: adapting and personalising medical care from the perspective of a refugee reception country
Source: Global Health. 2022 Oct 21;18:88. doi: 10.1186/s12992-022-00880-y (PMC9587623; doi:10.1186/s12992-022-00880-y)

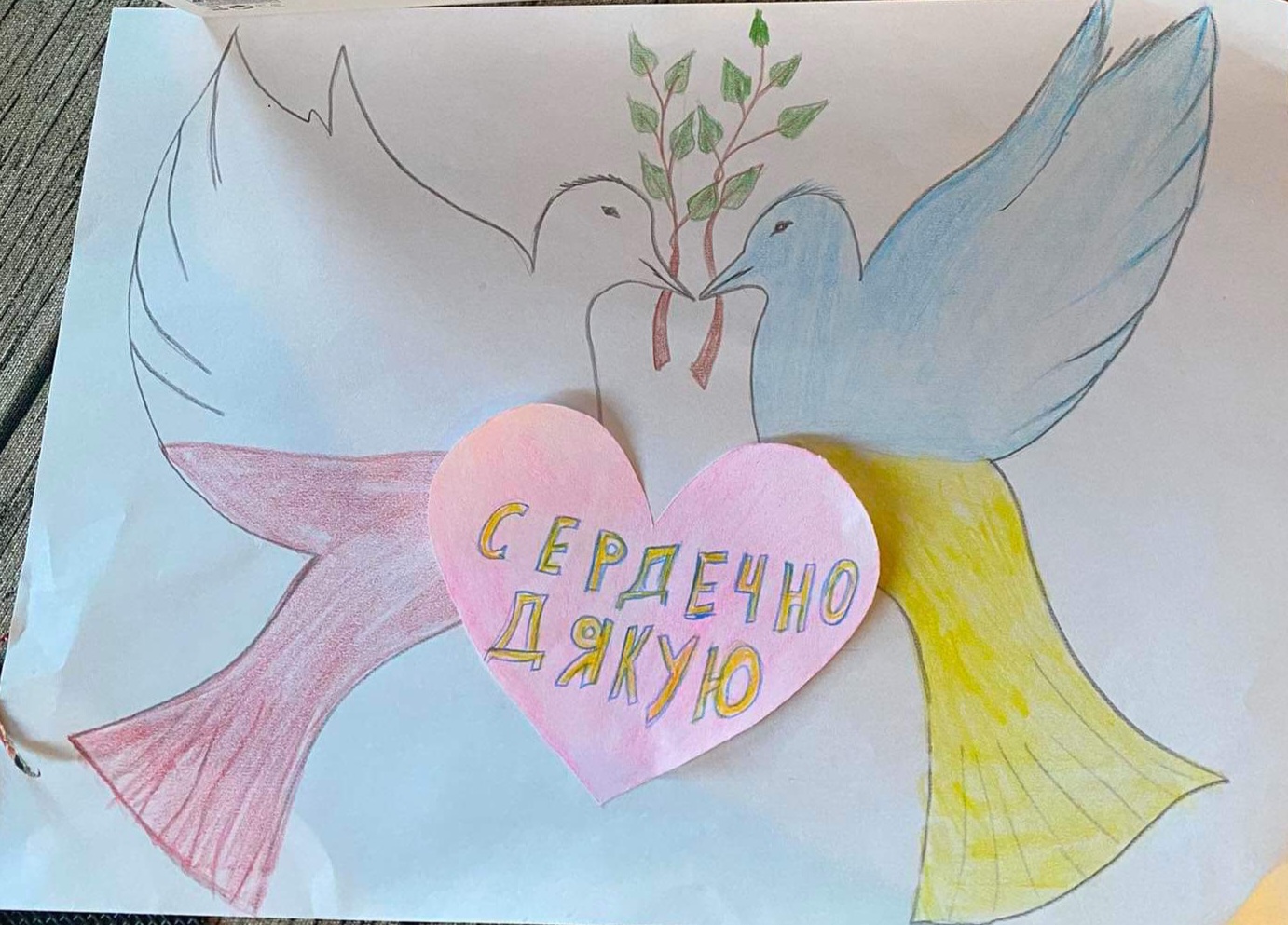

Supplement: Supplementary file 1 — Supplementary Material 1 [file 12992_2022_880_MOESM2_ESM.jpeg]

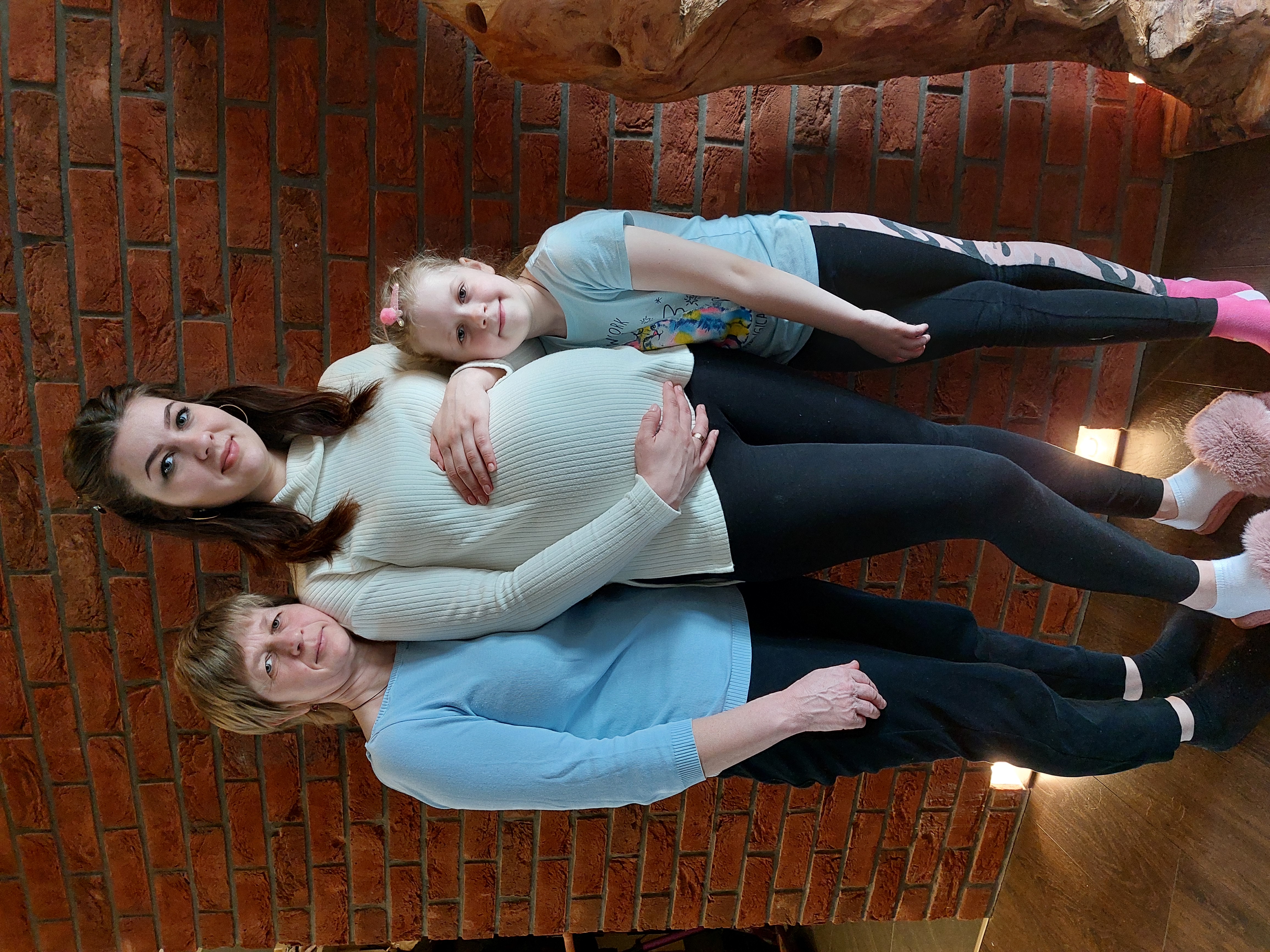

Supplement: Supplementary file 3 — Supplementary Material 3 [file 12992_2022_880_MOESM1_ESM.jpg]
