# Supplementary material for: A shared fate: adapting and personalising medical care from the perspective of a refugee reception country
Source: Global Health. 2022 Oct 21;18:88. doi: 10.1186/s12992-022-00880-y (PMC9587623; doi:10.1186/s12992-022-00880-y)
Supplement: Supplementary file 2 — Supplementary Material 2 [file 12992_2022_880_MOESM3_ESM.docx]

**Additional file 1.**

A photograph of a pregnant patient being removed from the hospital.

Maloletka E. UKR109 [photograph]. Associated Press; March 9, 2022. <https://www.apimages.com/metadata/Index/APTOPIX-Russia-Ukraine-War/31ca51dcead9440ca6fa9adc56e35b77/14/0> (Accessed 29 May 2022)
